# Roles of vitamin D in amyotrophic lateral sclerosis: possible genetic and cellular signaling mechanisms

**DOI:** 10.1186/1756-6606-6-16

**Published:** 2013-04-09

**Authors:** Khanh vinh quốc Lương, Lan Thi Hoàng Nguyễn

**Affiliations:** 1 Vietnamese American Medical Research Foundation, Westminster, CA, USA; 2 FACP, FACE, FACN, FASN, FCCP, and FACAAI (SC), 14971 Brookhurst St, Westminster, CA, 92683, USA

**Keywords:** Vitamin D, Calcitriol, Amyotrophic lateral sclerosis, ALS

## Abstract

Evidence suggests that there are aberrations in the vitamin D-endocrine system in subjects with amyotrophic lateral sclerosis (ALS). Here, we review the relationship between vitamin D and ALS. Vitamin D deficiency was reported in patients with ALS. Dietary vitamin D_3_ supplementation improves functional capacity in the G93A transgenic mouse model of ALS. Genetic studies have provided an opportunity to identify the proteins that link vitamin D to ALS pathology, including major histocompatibility complex (MHC) class II molecules, toll-like receptors, poly(ADP-ribose) polymerase-1, heme oxygenase-1, and calcium-binding proteins, as well as the reduced form of nicotinamide adenine dinucleotide phosphate. Vitamin D also exerts its effect on ALS through cell-signaling mechanisms, including glutamate, matrix metalloproteinases, mitogen-activated protein kinase pathways, the Wnt/β-catenin signaling pathway, prostaglandins, reactive oxygen species, and nitric oxide synthase.

In conclusion, vitamin D may have a role in ALS. Further investigation of vitamin D in ALS patients is needed.

## Introduction

Amyotrophic lateral sclerosis (ALS) is a fatal neurodegenerative disease that is characterized by progressive degeneration of motor neurons in the central nervous system, which results in muscle weakness, paralysis, and death. An abnormal calcium-parathyroid hormone (PTH)-vitamin D axis has been reported in patients with ALS. The serum concentration of 25-hydroxyvitamin D_3_ (25OHD) is significantly lower in ALS patients than in controls. Serum levels of PTH and ionized calcium are elevated in most ALS patients. The degree of hand grip dysfunction in ALS patients is also correlated with the Z score for metacarpal bone density [1]. Serum PTH levels have been shown to be mildly elevated in some ALS patients, and low levels of serum 25OHD have also been detected in ALS patients. In addition, significant positive correlations have been demonstrated between serum PTH levels and the duration of illness in male patients with motor neuron disease [2]. Recently, there are two papers that suggest a link between vitamin D and ALS [3,4]. Moreover, the gene encoding vitamin D-binding protein (DBP) (group-specific component, Gc) is a key factor for regulating calcium homeostasis through the vitamin D endocrine system and is a candidate contributor to susceptibility to osteoporosis in adult Japanese women [5]. DBP is a multifunctional protein that, in addition to the transport of vitamin D sterols, has a major role in the actin-scavenging system that removes globular monomeric actin (G-actin), which is released into the systemic circulation following cell damage [6,7]. The *Gc2* polymorphism of DBP was identified in the plasma of a group of Portuguese patients with familial ALS and was suggested as a risk factor for ALS [8]. Furthermore, an increased risk of ALS has been associated with occupational exposure to lead and with higher levels of both bone and blood lead [9]. In lead workers, the *VDR B* allele has been associated with an increase in blood and tibia lead levels [10,11]. In a rat model of peripheral nerve injury and repair, vitamin D_2_ significantly increases axogenesis and axon diameters, improves the responses of sensory neurons to metabolites such as KCl and lactic acid, and induces a fast-to-slow fiber type transition of the tibialis anterior muscle [12]. Dietary vitamin D_3_ supplementation at 10 times the adequate intake improves functional capacity in the G93A transgenic mouse model of ALS [13]. These findings suggest that vitamin D plays a role in ALS. Herein, we further discuss the potential role of vitamin D in ALS, along with possible genetic and cell signaling mechanisms.

## The genetic role of vitamin D in amyotrophic lateral sclerosis

### *The major histocompatibility complex (MHC) class II* molecule*s*

MHC class II molecules play an important role in the immune system and are essential in the defense against infection. Human MHC class II molecules are encoded by three different human leukocyte antigen (HLA) isotypes, *HLA-DR*, -*DQ*, and –*DP*. Studies have suggested that several genes in the MHC region promote susceptibility to ALS. In affected ALS tissue, reactive microglia stained prominently for HLA-DR, HLA-DP, and HLA-QD [14]. These areas include the primary motor cortex, the motor nuclei of the brain stem, the anterior horn of the spinal cord, and the full extent of the cortico-spinal tract. In the muscle of patients with ALS, most of the T-cells and macrophages that surround the atrophied muscle fibers are in an activated state, as indicated by their intense HLA-DR expression. In addition, some angulated degenerated fibers display strong endomysial positivity for HLA-DR in the regions in which T-cells and macrophages are present in clusters [15]. Increased expression of HLA-DR has been observed in the endoneurium of the peripheral nerves of ALS patients. The phenotypic characteristics of HLA-DR-positive cells are chiefly Schwann cells [16]. However, calcitriol inhibits differentiation, maturation, activation, and survival of dendritic cells and down-regulates MHC class II expression [17,18]. Calcitriol and its analogs modulate human dendritic cells by inhibiting HLA-DR expression [19]. The vitamin D analog ZK203278 potently inhibits lymphocyte proliferation in the mixed lymphocyte reaction and down-regulates MHC class II expression by 70% [20]. In addition, 1α-calcidol significantly modulates the expression of HLA-DR in human peripheral blood monocytes [21]. Intrinsic 25-OHD activation inhibits human dendritic cell antigen presentation and chemotaxis and reduces HLA-DR expression [22]. These findings suggest that calcitriol could have an effect on ALS through suppression of the expression of MHC class II antigens.

### Toll-like receptors (TLRs)

TLRs are a group of glycoproteins that function as surface trans-membrane receptors. These receptors are involved in the innate immune response to exogenous pathogenic micro-organisms. Chronic stimulation of innate immunity with endotoxin/lipopolysaccharide (LPS) has been shown to accelerate the disease course in superoxide dismutase-1 (SOD1) transgenic mice. However, ablation of proliferating microglia does not affect motor neuron degeneration in ALS caused by mutant SOD1 [23]. This finding suggests that proliferating microglia-expressing mutant SOD1 are not central contributors of the neurodegenerative process in ALS caused by mutant SOD1. Closely associated with the severity of disease is the stronger and restricted up-regulation of the receptor of innate immunity TLR-2 and pro-inflammatory cytokines in degenerating regions of the ventral spinal cord and in efferent fiber tracts of the brains of LPS-treated SOD1^G37R^ mice [24]. Up-regulation of TLR-2 has also been observed in ALS murine models [25], and the expression of an ALS-linked SOD1 mutant increases the neutotoxic potential of microglia via TLR-2 [26]. Increased LPS/TLR-4-signaling associated genes were observed in the peripheral blood mononuclear cells (PBMCs) from sporadic ALS patients after short-term cultivation, and elevated levels of gene expression correlated with the degree of peripheral blood monocyte activation and plasma LPS levels in sporadic ALS [27]. Immunohistochemical analysis of TLR-2 and TLR-4 revealed increased expression in reactive glial cells in both the gray (ventral horn) and white matter of ALS spinal cords. TLR-2 was predominantly detected in cells of the microglia/macrophage lineage, whereas TLR-4 was strongly expressed in astrocytes [28]. SH-SY5Y neuroblastoma cells transfected with the G93A mutant of SOD1 (which is typical of familial ALS) were more vulnerable to the neurotoxic action of pneumolysin and to the attack of monocytes stimulated by Pam3CSK4 and TLR-2 agonist than SH-SY5Y cells transfected with wild-type human SOD1 [29]. Calcitriol primes monocytes to respond less effectively to bacterial cell wall components in a VDR-dependent mechanism, due to decreased levels of TLR-2 and TLR-4 mRNA [30]. Calcitriol has also been shown to down-regulate intracellular TLR-2, TLR-4, and TLR-9 expression in human monocytes [31]. Interestingly, TLR activation up-regulates the expression of *VDR* and 1α-vitamin D hydroxylase in human monocytes [32]. Taken together, these data suggest that vitamin D plays a role in ALS via modulation of TLR pathways.

### Poly(ADP-ribose) polymerases (PARPs)

PARPs comprise a family of enzymes that share a conserved catalytic domain that support mono- or poly(ADP-ribosyl) transferase activity using NAD^+^ as a donor of ADP-ribosyl units. PARPs are involved in a wide range of molecular and cellular processes, including maintenance of genome stability, regulation of chromatin structure and transcription, cell proliferation, and apoptosis [33]. Expression of the DNA repair enzyme PARP occurs in response to oxidative DNA damage. In the ALS brain, PARP expression is increased in the motor cortex, parietal cortex, and cerebellum. PARP immunostaining in the motor cortex is increased in ALS neurons and subcortical glia and macrophages [34]. PARP expression is increased in astrocytes but is decreased in motor neurons in the spinal cord of sporadic ALS patients [35]. In the brainstem and cerebellum, PARP-immunoreactive astrocytes are observed in the medullary and pontine reticular formation, the hypoglossal nucleus, vestibular nucleus, cochlear nucleus, and cerebellar nuclei of SOD^G93A^ transgenic mice [36], suggesting that reactive astrocytes may play an important role in the pathogenesis and progression of ALS. Genetic disruption of the *PARP* gene provides profound protection against glutamate-nitric oxide-mediated ischemic insults *in vitro* and a major reduction in infarct volume after reversible middle cerebral artery occlusion [37]. These results provide compelling evidence for the primary involvement of PARP activation in neuronal damage following focal ischemia. Mice lacking the gene for *PARP* are dramatically less susceptible to 1-methyl-4-phenyl-1,2,3,6-tetrahydropyridine (MPTP) neurotoxicity [38]. The hAPP^J20^ mice, which accumulate amyloid β (Aβ) with ageing, develop microglial activation, reduced hippocampal CA1 calbindin expression, and impaired novel object recognition by the age of 6 months. All of these features are attenuated in hAPP^J20^/PARP-1^−/−^ mice. Similarly, the injection of Aβ^1-42^ into mouse brain produces a robust microglial response in wild-type mice that is blocked in mice that lack PARP-1 expression or activity [39]. Hydrogen peroxide-induced motor neuron apoptosis is prevented by the PARP inhibitors benzamide and nicotinamide [40]. These findings suggest the potential utility of PARS inhibitors in the treatment of neurodegenerative disorders such as ALS in which oxidative stress has been suspected to play an important role. However, treatment of transgenic ALS mice with PARP inhibitors resulted in a non-significant trend toward increased survival [41]. Furthermore, treatment with dexamethasone and vitamin D_3_ attenuates neuroinflammatory age-related changes in the rat hippocampus; caspase-3 and PARP were all attenuated in hippocampal tissue prepared from rats that received dexamethasone and vitamin D_3_[42]. Increased levels of vitamin D appear to down-regulate PARP-1 expression; PARP-1 levels decrease following calcitriol treatment in NB4 cells, which are acute promyelocytic leukemia cells [43]. Vitamin D exerts a concentration-dependent inhibitory effect on PARP-1 in human keratinocyte cells [44]. These findings suggest that vitamin D may have a protective role in ALS by down-regulating PARP.

### Heme oxygenase-1 (HO-1)

HO-1 is a stress protein that can confer cytoprotection by enhancing the catabolism of pro-oxidant heme to the radical scavenging bile pigments biliverdin and bilirubin. In various models of oxidative tissue injuries, the induction of HO-1 protects tissues from further damage via heme removal [45]. In rat astrocytes, human HO-1 over-expression results in significant oxidative damage to mitochondrial lipids, proteins, and nucleic acids and increased cell death [46]. HO-1 induction in the motor cortex has been reported in TDP-43 A315T transgenic mice, which develop degeneration of specific motor neurons. The accumulation of ubiquitinated proteins has been observed in the pyramidal cells of the motor cortexes of these mice [47]. In SOD1^G93A^ transgenic mice, the *SOD1-G93A* transgene and HO-1 are preferentially over-expressed in the lumbar spinal cord, particularly in activated astrocytes [48]. In the spinal motor neurons of ALS model mice, expression levels of HO-1 displayed a progressive increase but were significant only in the surrounding glial cells at 18 weeks [49]. However, pretreatment with vitamin D_3_ ameliorates systemic IL-6 levels following ischemia and reperfusion of bilaterally occluded vessels in rats, improves lung and muscle injury, and results in a significant increase in leukocyte HO-1 expression [50]. Moreover, following the focal cortical ischemia that is elicited by photo-thrombosis, calcitriol treatment results in a transient but significant up-regulation of glial HO-1 immunoreactivity. This up-regulation is concomitant with a reduction in glial fibrillary acidic protein (GFAP) in remote cortical regions affected by the secondary spread of injury in glial cells [51]. These results support the protective role of calcitriol in post-cellular injury.

### Calcium-binding proteins

In ALS, altered calcium homeostasis appears to contribute significantly to selective neuronal injury. At least three calcium-binding proteins are abundant in various types of nerve cells: calbindin-D_28K_, calretinin, and parvalbumin. In the rat cerebellar cortex, calbindin-D_28K_ mRNA was detected in the Purkinje cells, and parvalbumin mRNA was located in the Purkinje cells as well as in the basket/stellate cells of the molecular layer. Calretinin, by contrast, was found only in the granule cell layer [52]. Reduced calreticulin levels link endoplasmic reticulum stress and Fas-triggered cell death in motor neurons that are vulnerable to ALS [53]. Calbindin-D_28K_ and/or parvalbumin appear to influence the selective vulnerability of motor neurons in ALS. Parvalbumin has been suggested as a marker of ALS-resistant motor neurons [54]. In human autopsy specimens, immunoreactive calbindin-D28K and parvalbumin are absent in motor neuron populations that are lost early in ALS (i.e., cortical and spinal motor neurons, lower cranial nerve motor neurons) [55]. In transgenic mice, parvalbumin-positive anterior horn neurons are severely reduced, even at the presymptomatic stage, whereas calbindin-positive neurons are largely preserved. At the symptomatic stage, both parvalbumin and calbindin-D_28K_ immunoreactivity markedly diminish or disappear in the anterior horn. Immunoblotting analysis revealed a significant reduction of immunoreactivity to the parvalbumin antibody in transgenic mice compared with controls [56]. In the cerebral cortex of SOD1^G93A^ transgenic mice, the number and staining intensity of calretinin-positive neurons is decreased. In the hippocampal formation, layer-specific alterations in the staining intensity of calretinin-immunoreactive neurons are observed in the CA1-3 areas and the dentate gyrus [57]. Over-expression of parvalbumin in transgenic mice rescues motor neurons from injury-induced cell death [58]. Parvalbumin transgenic mice interbred with mutant SOD1 transgenic mice display significantly reduced motor neuron loss, delayed disease onset (17%), and prolonged survival (11%) when compared with mice with only the mutant SOD1 transgene. Increased motor neuron parvalbumin can significantly attenuate the immune-mediated increases in calcium and, to a lesser extent, compensate for the mutant SOD1-mediated toxic-gain-of-function in transgenic mice [59]. Treatment with calbindin-D_28K_ antisense oligodeoxynucleotides that significantly decrease calbindin-D_28K_ expression rendered these cells vulnerable again to ALS IgG toxicity [60]. Overexpression of melatonin membrane receptors increases calcium-binding proteins, calbindin-D_28K,_ and parvalbumin and protects VSC4.1 motor neurons from glutamate toxicity through multiple mechanisms [61]. Moreover, calbindin-D_28K_ is a 1 α,25-dihydroxyvitamin D_3_-induced calcium-binding protein [62]. Human syncytiotrophoblast cells express calbindin-D_9k_ and calbindin-D_28K_ genes, which are stimulated by calcitriol [63]. Parvalbumin increases in the caudate putamen of rats with vitamin D hypervitaminosis [64], which suggests that the metabolism of parvalbumin in the caudate putamen can be influenced by variations in the blood level of vitamin D. Calcitriol induces a two-fold increase in the immunoreactivity for calbindin-D_28K_ and parvalbumin in motor neuron cells. Injection of 80–120 ng calcitriol in the cerebral ventricles of adult rats also induced positive immunoreactivity for calcium binding proteins in ventral motor neurons [65], suggesting that calcitriol might be a useful tool for enhancing the expression of calcium binding proteins in the motor system and has potential therapeutic value for neurodegenerative disease.

### The reduced form of the nicotinamide adenine dinucleotide phosphate (NADPH) oxidase (Nox) enzyme complex

Nox mediates critical physiological and pathological processes, including cell signaling, inflammation, and mitogenesis, by generating reactive oxygen species (ROS) from molecular oxygen. Protein disulfide isomerase (PDI) in ALS mouse glia links protein misfolding with Nox-catalyzed superoxide (O_2_^-^) production. Inhibition of PDI or its down-regulation by short interfering RNAs prevents Nox activation in microglia and subsequent production of O_2_^-^[66]. SOD1 directly regulates Nox-dependent O_2_^-^ production by binding Rac1 and inhibiting its GTPase activity. Glial cell toxicity associated with the expression of SOD1 mutants in culture is significantly attenuated by treatment with the Nox inhibitor apocynin. Treatment of ALS mice with apocynin also significantly increases their average life span [67]. Deletion of either Nox gene significantly slows disease progression and improves survival. However, 50% survival rates were enhanced significantly more by Nox2 deletion than by Nox1 deletion. Interestingly, female ALS mice that contained only 1 active X-linked Nox1 or Nox2 gene also display significantly delayed disease onset but normal disease progression rates [68]. Mesenchymal stem cell (MSC) transplantation in the lumbar spinal cord prolongs survival in a rat model of ALS. The intra-thecal delivery of MSCs and the subsequent generation of healthy astrocytes at a symptomatic stage decreases motor neuron loss, preserving motor functions and extending the survival of rats expressing human SOD1^G93A^. This neuroprotection is correlated with decreased inflammation, as shown by the lower proliferation of microglial cells and the reduced expression of Nox2 in the lumbar spinal cord [69]. However, vitamin D deprivation in rats decreases the activity of cytosolic NADPH-dependent 3,5,3′-triodo-L-thyronine (T_3_) binding in the liver. This decrease can be restored by administering calcitriol [70]. In heart mitochondria, NAD^+^-dependent isocitrate dehydrogenase is notably decreased in vitamin D-deficient rats, but treatment with calcitriol restores normal values [71]. In rat centrilobular hepatocytes, a vitamin D-deficient diet induces a significant increase in NADPH [72]. Taken together, these findings and results indicate that vitamin D may play a role in ALS via the suppression of NADPH expression.

## The non-genomic role of vitamin D in ALS

Neurodegeneration disease is the umbrella term for the disease progressive loss of structure or function of neurons, including death of neurons. Many neurodegenerative diseases including Alzheimer’s, Parkinson’s, and ALS diseases occur as a result of neurodegenerative processes. As research progresses, many similarities appear which relate these diseases to one another on a sub-cellular level. Interestingly, vitamin D was shown to have a role in these neurodegenerative diseases [73,74].

### Neurotransmitter Glutamate

Glutamate is an excitatory neurotransmitter in the central nervous system and has been suggested to play a major role in ALS. Abnormalities glutamate concentrations have been identified by proton magnetic resonance spectroscopy in the brain and spinal cord of rodent FALS1 models, with changes in glutamine levels [75,76]. Increased plasma glutamate levels are observed in ALS and are correlated with longer disease duration and male gender [77]. Plasma glutamate levels are significantly elevated (by approximately 70%) in ALS patients compared with controls. By contrast, glutamate levels are significantly decreased in all CNS regions studied in ALS patients (by 21-40%), with the greatest changes occurring in the spinal cord. The ratio of glutamine to glutamate is altered significantly in spinal cord ALS tissue [78]. A 43% reduction of the high-affinity glutamate uptake rate has been observed in patients with ALS compared with normal controls and chronic neurological disorder patients [79], suggesting a systemic impairment of glutamate uptake in ALS. In patients with ALS, a marked decrease in the maximal velocity of transport for high-affinity glutamate uptake in synaptosomes from the spinal cord (−59%), motor cortex (−70%), and somatosensory cortex (−39%), but not in those from the visual cortex, striatum, or hippocampus, has been observed [80]. Platelets of ALS patients displayed a 37% increase in the expression of glutamine synthetase but a normal expression of glutamate transporter [81]. However, the only treatment that is approved for use in patients with ALS is the anti-glutamate drug Riluzole. Daily 100 mg oral consumption of the drug prolongs the median survival of patients by approximately 2–3 months and increases the likelihood of survival in the first year by 9%. Although modestly effective at best, the drug acts as a voltage-dependent sodium channel blocker while also inhibiting glutamate release from the presynaptic terminal and increasing glutamate re-uptake into the surrounding astrocytes [82]. Furthermore, several *in vivo* and *in vitro* studies have demonstrated the neuroprotective potential of pretreatment with calcitriol. Combinatorial treatment with progesterone and 1,25-dihydroxyvitamin D_3_ produces better neuroprotection than progesterone alone following glutamate-induced excitotoxic neuronal injury *in vitro*[83]. Calcitriol protects dopaminergic neurons against cytotoxicity induced by glutamate and dopaminergic toxins by facilitating cellular functions that reduce oxidative stress in a mesencephalic culture [84]. Calcitriol was neuroprotective when it was administered together with glutamate or even after a delay of up to 6 hours during a 24-hour excitotoxic challenge of hippocampal and neocortical cells. In addition, calcitriol reduces glutamate-induced caspase-3 activity in cerebellar granule cells dependent on cell maturity [85]. Chronic vitamin D_3_ treatment protects against neurotoxicity by glutamate in association with up-regulation of vitamin D receptor mRNA expression in cultured rat cortical neurons [86]. Taken together, vitamin D may play a role in ALS by reducing glutamate-induced neurotoxicity.

### *Matrix metalloproteinases* (MMPs)

MMPs are proteolytic enzymes that are responsible for remodeling the extracellular matrix and regulating leukocyte migration through the extracellular matrix. This migration is an important step in inflammatory and infectious pathophysiology. MMPs are produced by many cell types, including lymphocytes, granulocytes, astrocytes, and activated macrophages. There is growing evidence that MMPs play an important role in the pathogenesis of ALS. Immunohistochemical studies have established the presence of MMP-2 in astrocytes and MMP-9 in pyramidal neurons in the motor cortex and motor neurons in the spinal cord of ALS patients [87]. MMP-2 and MMP-9 are elevated in the sera, cerebrospinal fluid, spinal cord, and skin of patients with ALS and in a mouse model of ALS [88-91]. Pro and active MMP-9 are elevated in the sera of ALS patients compared with healthy controls. Pro-MMP-9 is elevated in the sera as well as in extracts of damaged nerve and muscle of ALS [92], which suggests that such damage might be followed by elevated pro-MMP-9 in sera. MMP-9 deficiency in G93A mice significantly attenuates neuronal loss, reduces neuronal TNF-alpha and FasL immunoreactivities in the lumbar spinal cord, and increases survival (31%) [93], which suggests that MMP-9 contributes to motor neuron cell death in ALS. Treatment with an MMP inhibitor starting at 30 days of age improved motor performance and significantly prolonged the survival time of the animals; however, administration at disease onset did not significantly improve the survival time [94]. These findings suggest that early pharmacological inhibition with a synthetic MMP inhibitor extended the survival of the animals, which suggests a role for MMPs in the early phase of the disease. Moreover, VDR-knock-out mice have been shown to exhibit an influx of inflammatory cells, phospho-acetylation of NF-κB, and up-regulated expression of MMP-2, MMP-9, and MMP-12 in the lungs [95]. The *VDR TaqI* polymorphism is associated with the decreased production of TIMP-1, a natural MMP-9 inhibitor [96]. In addition, calcitriol modulates tissue MMP expression under experimental conditions [97], Calcitriol also inhibits both basal levels and staphylococcal-stimulated production of MMP-9 in human blood monocytes and alveolar macrophages [98]. Together, these studies suggest that calcitriol might play an important role in the pathological processes of ALS by down-regulating the level of MMPs and regulating the level of TIMPs.

### *The mammalian family of mitogen-activated protein kinases* (MAPKs)

MAPKs includes extracellular signal-regulated kinase (ERK), p38, and c-Jun NH_2_-terminal kinase (JNK), with each MAPK signaling pathway comprising at least three components, a MAPK 3 kinase (MAP3K), a MAPK 2 kinase (MAP2K), and a MAPK. The MAPK pathways are activated by diverse extracellular and intracellular stimuli, including peptide growth factors, cytokines, hormones, and various cellular stressors, such as oxidative stress and endoplasmic reticulum stress. These signaling pathways regulate a variety of cellular activities, including proliferation, differentiation, survival, and death [99]. Activated p38MAPK is a novel component of the intracellular inclusions that are found in human ALS and mutant SOD1 transgenic mice [100], which suggests that activation of p38MAPK might contribute significantly to the pathology of motor neurons in ALS. Activation of the stress-activated p38MAP kinase but not JNK in cortical motor neurons is observed during early presymptomatic stages of ALS in transgenic mice [101]. Accumulation of p38MAPK has been detected by immunoblotting in the spinal cord of G93A mice during the progression of the disease. As the disease progresses, activated p38MAPK also accumulates in hypertrophic astrocytes and reactive microglia [102]. These findings suggest that persistent activation of p38 mitogen-activated protein kinase in a mouse model of familial ALS correlates with disease progression. Activation of the p38MAPK cascade is associated with up-regulation of TNFα receptors in the spinal motor neurons of mouse models of familial ALS [103]. The p38MAPK-inhibitor SB203580 completely inhibits mutant SOD1-induced apoptosis of motor neurons and blocks LPS-induced activation of microglia. Semapimod, a p38MAPK inhibitor suitable for clinical use, prolongs the survival of mutant SOD1 mice to a limited extent but largely protects motor neurons and proximal axons from mutant SOD1-induced degeneration [104]. By regulating *VDR* mRNA expression, the p38 MAPK pathway participates in the mediation of calcium signals and affects lipid accumulation in murine pre-adipocytes [105]. Pretreatment with calcitriol has been shown to inhibit JNK activation by all stressors and to inhibit p38 activation in keratocytes [106]. Zhang et al. [107] demonstrated that the up-regulation of MKP-1 by vitamin D inhibits LPS-induced p38 activation and cytokine production in monocytes and macrophages. Another study demonstrated that the vitamin D analog (24R)-1,24-dihydroxycholecalciferol prevents neuronal damage caused by hydrogen peroxide-induced toxicity in the SH-SY5Y cell line [108]. Interestingly, the neurotoxic effects of H_2_O_2_ are dependent on JNK and p38 MAPK. Taken together, these results suggest that vitamin D might play a role in ALS by modulating MAPK.

### The Wnt/β-catenin signaling pathway

Wnt/β-catenin plays a pivotal role in regulating cell growth, cell development, and the differentiation of normal stem cells. Various Wnts are expressed in the developing CNS and peripheral nervous system [109]. Wnt/β-catenin signaling is implicated in determining the balance between neuronal survival and death in a variety of neurodegenerative diseases [110-112]. Wnt2 and Wnt7a mRNA and protein are up-regulated in the spinal cord of ALS mice compared with wild-type mice. Moreover, the immunoreactivity of Wnt2 and Wnt7a is strong in ALS mice but weak in wild-type mice at the same time points. Double immunofluorescence labeling demonstrated that Wnt2 and Wnt7a are expressed in both neurons and astrocytes [113]. The mRNA and protein levels of Wnt3a and cyclin D1 in the spinal cords of ALS mice are up-regulated compared to those in wild-type mice. In addition, β-catenin translocates from the cell membrane to the nucleus and subsequently activates the transcription of its target gene, cyclin D1. Moreover, Wnt3a, β-catenin, and cyclin D1 are also expressed in both neurons and astrocytes. The expression of Wnt3a, β-catenin, or cyclin D1 is increased in mature GFAP^+^ astrocytes [114]. These findings suggest that neurodegeneration activates the Wnt/β-catenin signaling pathway, which is associated with glial proliferation in the adult spinal cord of ALS transgenic mice. However, calcitriol inhibits β-catenin transcriptional activity by promoting the binding of β-catenin and VDR and the induction of E-cadherin expression [115]. Most VDR variants fail to activate the vitamin D-responsive promoter and fail to bind β-catenin or regulate its activity [116]. Taken together, vitamin D has a role in ALS by modulating the Wnt/β-catenin signaling pathway.

### *Prostaglandins* (PGs)

Play a role in inflammatory processes. Cyclooxygenase (COX) participates in the conversion of arachidonic acid into PGs. PGE_2_ activity is associated with motor neuron death through the induction of free radical formation and glutamate release from astrocytes. Serum and cerebrospinal fluid (CSF) PGE_2_ concentrations are significantly higher in ALS patients compared with controls [117]. Levels of the potent PGE_2_ are elevated in post-mortem spinal cords from patients with ALS [118]. CSF PGE_2_ levels are markedly increased in ALS specimens compared to non-ALS specimens, and COX-2 expression is dramatically increased in the spinal cords of patients with ALS. The COX-2 protein is found in motor neurons, interneurons, and glial cells [119]. The PGE_2_ receptor (EP_2_) is significantly induced in SOD^G93A^ mice in astrocytes and microglia in parallel with increases in the expression of pro-inflammatory enzymes and lipid peroxidation. In human ALS, EP_2_ immunoreactivity is up-regulated in astrocytes in the ventral spinal cord. In aging SOD^G93A^ mice, genetic deletion of EP_2_ improves motor strength and extends survival. Deletion of EP_2_ in SOD^G93A^ mice results in significant reductions in levels of pro-inflammatory effectors, including COX-1, COX-2, inducible nitric oxide synthase (iNOS), and components of the Nox complex [120]. These data suggest that PGE_2_ signaling via EP_2_ functions in the mutant SOD model and more broadly in inflammatory neurodegeneration to regulate the expression of a cassette of pro-inflammatory genes. The level of microsomal PG synthase-1 (mPGES-1) was increased in SOD^G93A^ mice at 15 weeks and older [121]. In SOD^G93A^ transgenic mice, AAD-2004, 2-hydroxy-5-[2-(4-trifluoromethylphenyl)-ethylaminobenzoic acid] blocked free radical production and PGE_2_ formation and inhibits mPGES1 and microglial activation in the spinal cord. As a consequence, AAD-2004 reduces auto-phagosome formation, axonopathy, and motor neuron degeneration, improving motor function and increasing life span [122]. These results suggest that mPGES-1 in motor neurons could play a role in the pathogenesis of ALS and that mPGES-1 could work sequentially in motor neurons and activated microglia to produce ALS symptoms in SOD^G93A^ mice. COX-2 mRNA is up-regulated 7.09-fold in ALS compared with non-ALS spinal cords [123]. Intra-thecal delivery of MSCs decreases motor neuron loss, preserves motor functions, and extends the survival of human SOD1^G93A^ rats. This neuroprotection is correlated with decreased inflammation, as evidenced by the reduced proliferation of microglial cells and the reduced expression of COX-2 in the lumbar spinal cord [69]. Increased expression of neuronal COX-2 has been observed in the hippocampi of ALS patients both with and without dementia [124]. The level of COX-2 is up-regulated in microglia and astrocytes by CD40 stimulation in vitro. CD40 stimulation in primary spinal cord cultures causes motor neuron loss that is protected by a selective COX-2 inhibitor [125]. Treatment with a selective cyclooxygenase-2 inhibitor, celecoxib, markedly inhibits production of PGE_2_ in the spinal cords of ALS mice. Celecoxib treatment significantly delays the onset of weakness and weight loss and prolongs survival by 25%-30%. Spinal cords of treated ALS mice displayed significant preservation of spinal neurons and diminished astrogliosis and microglial activation [126]. Oral administration of the non-selective COX inhibitor sulindac extends survival by 10% in SOD1^G93A^ mice compared to littermate controls. Sulindac, as well as the selective COX-2 inhibitors rofecoxib and celecoxib, reduces cPLA2 immunoreactivity in the lumbar spinal cord of G93A transgenic mice. Sulindac treatment preserves motor neurons and reduces microglial activation and astrocytosis in the spinal cord [127], suggesting that cPLA2 plays an important role in supplying arachidonic acid to the COX-2 driven inflammatory pathway in ALS associated with SOD1 mutations. Moreover, the combination of relatively COX-2 selective molecules and aspirin is associated with higher bone mineral density at multiple skeletal sites in men and women compared to controls [128]. Calcitriol is reported to regulate the expression of several key genes involved in the PG pathway, resulting in a decrease in PG synthesis [129]. Calcitriol pretreatment limits PG biosynthesis by cytokine-stimulation in adult human osteoblast-like cells [130]. Calcitriol and its analogs have been shown to selectively inhibit the activity of COX-2 [131]. Calcitriol induces NAD^+^-dependent 15-hydroxyprostaglandin dehydrogenase (15-PGDH) in human neonatal monocytes. In addition, COX-2 mRNA and PGE_2_ levels were decreased in the culture medium compared to controls [132]. 15-PGDH is the key enzyme of PGE_2_ catabolism. In addition, PGE_1_ increases *in vivo* and *in vitro* calcitriol biosynthesis in rabbits [133]. These findings suggest that vitamin D might play a role in modulating the inflammatory process in ALS.

### Reactive oxidative stress (ROS)

ROS are produced by activated phagocytes as a part of their microbicidal activities. Oxidative stress biomarkers, urinary 8-oxodeoxyguanosine, and urinary 15-F_2t_-isoprostane have been demonstrated in sporadic ALS [134]. Increased ROS have been noted in familial ALS with mutations in SOD1 [135]. Compared with controls, the levels of H_2_O_2_ and the hydroxyl radical are significantly higher and the level of O_2_^-^ is significantly lower in ALS mutant mice [136]. Approximately 20% of familial ALS is associated with mutations in the gene for SOD1, which is encoded on chromosome 21q22.1. A decrease in Cu/Zn- and Mn-SOD activity has been demonstrated in the brains and spinal cords of patients with ALS [137,138]. These low levels increase the production of ROS and cause mitochondrial damage and death in motor neuron-like cells [138]. Overexpression of MnSOD or SOD2 attenuates neuronal death in human cells that express mutant (G37R) Cu/Zn-SOD [139,140]. SOD2 overexpression also markedly attenuates the neuronal toxicity induced by adenovirus-mediated expression of all four SOD1 mutants. A significant increase in mitochondrial O_2_^-^ levels in neural cells that express mutant SOD1 has been observed. These elevated O_2_^-^ levels in mitochondria were significantly diminished by the overexpression of SOD2 [140]. These data suggest that mitochondrial-produced O_2_^-^ radicals play a critical role in mutant SOD1-mediated neuronal toxicity and implicate mitochondrial-produced free radicals. Increased reduction of oxidized glutathione (GSH) in CSF from patients with the sporadic form of ALS has been observed [141]. In an ALS-like transgenic mouse model, the reduction of GSH in the spinal cord and motor neuron cells is correlated with apoptosis-inducing factor translocation, caspase-3 activation, and motor neuron degeneration during ALS-like disease onset and progression [142], suggesting that decreased GSH promotes multiple apoptotic pathways that contribute to motor neuron degeneration in ALS. Similarly, calcitriol has been reported to exert a receptor-mediated effect on the secretion of H_2_O_2_ by human monocytes [143]. Human monocytes in culture gradually lose their capacity to produce O_2_^-^ when stimulated. The addition of calcitriol, LPS, or lipoteichoic acid restores the capacity of stimulated monocytes to produce O_2_^-^ and increases their oxidative capacity compared with unstimulated monocytes [144]. Calcitriol can also protect nonmalignant prostate cells from oxidative stress-induced cell death by eliminating ROS-induced cellular injuries [145]. Vitamin D metabolites and vitamin D analogs have been reported to induce lipoxygenase mRNA expression, lipoxygenase activity, and ROS in a human bone cell line [146]. In another study, the vitamin D analog (24R)-1,24-dihydroxycholecalciferol prevented neuronal damage caused by H_2_O_2_-induced toxicity in the SH-SY5Y cell line [108]. Vitamin D can also reduce the extent of lipid peroxidation and can induce SOD activity in a hepatic anti-oxidant system in rats [147]. Astrocytes play a pivotal role in the CNS detoxification pathways, in which GSH is involved in eliminating oxygen and nitrogen reactive species such as nitric oxide (NO). Calcitriol affects this pathway by enhancing intracellular GSH pools and significantly reduces the nitrite production that is induced by LPS [148]. These findings suggest that vitamin D modulates oxidative stress in ALS.

### Nitric oxide (NO)

**Figure 1:**
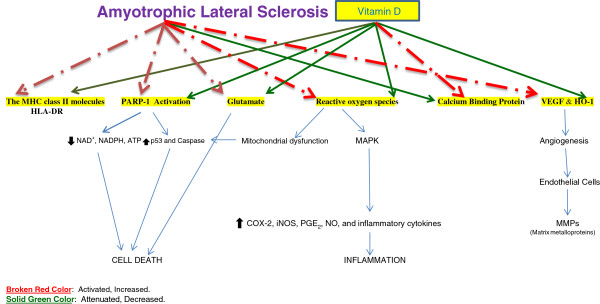
Illustrates the beneficial role of vitamin D in ALS.

The enzyme NOS is involved in the synthesis of NO, which regulates a variety of important physiological responses, including cell migration, the immune response, and apoptosis. An increase in oxidized NO products has been reported in the CSF of patients with the sporadic form of ALS [141]. Normal SOD1 increases the measured NO release *in vitro*, whereas cells expressing mutant SOD1 released less NO. Co-administration of two different NOS inhibitors (L-NAME and L-N-methyl arginine) resulted in a partial neuroprotective effect [149], suggesting that NO is likely to contribute to motor neuron injury. However, this observation does not fully account for all of the cellular toxic effects of mutant SOD1. iNOS mRNA levels and catalytic activity are increased significantly in the spinal cords of these transgenic mSOD1 mice [150]. Conversely, the activation of 1*α*-hydroxylase in macrophages increases the level of calcitriol, which inhibits iNOS expression and reduces NO production within LPS-stimulated macrophages [151]. Thus, calcitriol production by macrophages can provide protection against the oxidative injuries caused by the NO burst. Calcitriol is known to inhibit LPS-induced immune activation in human endothelial cells [152]. In experimental allergic encephalomyelitis, calcitriol inhibits the expression of iNOS in the rat CNS [153]. The arteries of vitamin D-deficient offspring have an impaired ability to relax because of deficiencies in the production of two important factors, NO and endothelium-derived hyperpolarizing factor. Vitamin D-deficient female offspring display additional impairment in the NO signaling pathway in the arterial muscle [154] Figure 1.

## Conclusions

This paper reviewed the relationship between vitamin D and ALS. Genetic studies have provided opportunities to identify the proteins that link vitamin D to ALS pathology. Vitamin D has a useful effect in ALS by suppressing of the expression of MHC class II antigens, enhancing TLRs, down-regulating PARP-1, up-regulating HO-1 expression, enhancing the expression of calcium-binding protein, and suppressing of NADPH expression. Vitamin D can also act through many non-genomic mechanisms, Vitamin D also exerts its effect on ALS through cell-signaling mechanisms, including glutamate, matrix metalloproteinases, mitogen-activated protein kinase pathways, the Wnt/β-catenin signaling pathway, prostaglandins, reactive oxygen species, and nitric oxide synthase Therefore, further investigation of vitamin D in ALS patients is needed.

## Competing interests

The authors declare that they have no competing interests.

## Authors’ contributions

This work was carried out in collaboration between both authors. Author KL designed the study and wrote the protocol. Author LN managed the literature searches. Both authors read and approved the final manuscript.
